# Homeostatic structural plasticity can account for topology changes following deafferentation and focal stroke

**DOI:** 10.3389/fnana.2014.00115

**Published:** 2014-10-16

**Authors:** Markus Butz, Ines D. Steenbuck, Arjen van Ooyen

**Affiliations:** ^1^Simulation Lab Neuroscience - Bernstein Facility for Simulation and Database Technology, Institute for Advanced Simulation, Jülich Aachen Research Alliance, Forschungszentrum JülichJülich, Germany; ^2^Student of the Medical Faculty, University of FreiburgFreiburg, Germany; ^3^Department of Integrative Neurophysiology, VU University AmsterdamAmsterdam, Netherlands

**Keywords:** topology, deafferentation, focal retinal lesion, neuronal network model, structural plasticity, homeostatic plasticity, stroke, epileptogenesis

## Abstract

After brain lesions caused by tumors or stroke, or after lasting loss of input (deafferentation), inter- and intra-regional brain networks respond with complex changes in topology. Not only areas directly affected by the lesion but also regions remote from the lesion may alter their connectivity—a phenomenon known as diaschisis. Changes in network topology after brain lesions can lead to cognitive decline and increasing functional disability. However, the principles governing changes in network topology are poorly understood. Here, we investigated whether homeostatic structural plasticity can account for changes in network topology after deafferentation and brain lesions. Homeostatic structural plasticity postulates that neurons aim to maintain a desired level of electrical activity by deleting synapses when neuronal activity is too high and by providing new synaptic contacts when activity is too low. Using our Model of Structural Plasticity, we explored how local changes in connectivity induced by a focal loss of input affected global network topology. In accordance with experimental and clinical data, we found that after partial deafferentation, the network as a whole became more random, although it maintained its small-world topology, while deafferentated neurons increased their betweenness centrality as they rewired and returned to the homeostatic range of activity. Furthermore, deafferentated neurons increased their global but decreased their local efficiency and got longer tailed degree distributions, indicating the emergence of hub neurons. Together, our results suggest that homeostatic structural plasticity may be an important driving force for lesion-induced network reorganization and that the increase in betweenness centrality of deafferentated areas may hold as a biomarker for brain repair.

## 1. Introduction

Repair of brain networks following lesions, stroke or neurodegeneration goes along with massive rewiring of connections. Rewiring is brought about by synapse formation and deletion, dendritic remodeling, and axonal sprouting, pruning and re-routing (structural plasticity) (Butz et al., 2009b). Network rewiring induced by lesions or neuronal loss contributes to changes in network topology associated with tumors (Bartolomei et al., 2006; Honey and Sporns, 2008), stroke (van Meer et al., 2012; Yin et al., 2013), and neurodegenerative diseases, including Alzheimer's disease (Stam et al., 2009; Sanz-Arigita et al., 2010) and multiple sclerosis (He et al., 2009; Tewarie et al., 2014). Interestingly, in all these pathologies, brains become more randomly connected or lose complexity of hierarchical structure (Tewarie et al., 2014). Increasing randomness and decreasing betweenness centrality (a topological measure for the importance of neurons in a network) correlate with network degeneration and decline in cognitive function (Bosma et al., 2009; Schoonheim et al., 2013). An important aspect of network rewiring is diaschisis (von Monakov, 1914; Andrews, 1991), the phenomenon that brain regions not directly affected by the primary lesion but deafferentated by the lesion change their connectivity. Extending this early concept of diaschisis, recent studies analysing neuroimaging data (e.g., from stroke patients) using graph theoretical methods have revealed complex changes in global network topology after brain lesions (Honey and Sporns, 2008; Alstott et al., 2009; Carter et al., 2012; van Meer et al., 2012; Rehme and Grefkes, 2013). These studies showed that while brain networks as a whole generally become more random following network rewiring, the deafferentated areas themselves increase their betweenness centrality (Wang et al., 2010)—an unexpected result because random networks tend to have nodes with low betweenness centrality. Changes in topology after brain damage have mostly been reported for inter-area connectivity (Wang et al., 2010), but both global inter-area connectivity and local intra-area connectivity rewire after lesions (Murphy and Corbett, 2009; Winship and Murphy, 2009).

Topology changes in inter-area and intra-area connectivity are poorly understood, partly because of a lack of understanding of the principles governing structural plasticity. An elegant way to study structural plasticity after deafferentation is the experimental paradigm of focal retinal lesions (Eysel et al., 1980; Keck et al., 2008; Yamahachi et al., 2009). In this paradigm, the primary lesion is made in the eye so that no damage of brain tissue overlays the massive cortical reorganization following deafferentation (Darian-Smith and Gilbert, 1994; Keck et al., 2008; Yamahachi et al., 2009; Keck et al., 2011; Marik et al., 2014). Compared with the brain, the eye is also better accessible for lesioning, and because of retinotopy, the retinal lesion leads to a well-defined deafferentated lesion projection zone (LPZ) in the primary visual cortex. Recently, we postulated that the need of neurons to maintain homeostasis of their average electrical activity may act as a driving force for structural plasticity (Butz and van Ooyen, 2013) (see also van Ooyen and van Pelt, 1994; van Ooyen et al., 1995; Butz et al., 2008, 2009a; Tetzlaff et al., 2010; van Ooyen, 2011). We developed a novel computational model, called Model of Structural Plasticity (MSP) (Butz and van Ooyen, 2013; Butz et al., 2014), in which neurons create new dendritic spines and axonal boutons when neuronal activity is below a homeostatic set-point, and delete spines and boutons when activity is above the set-point. Synapses are formed by merging spines and boutons. Using MSP, we showed (Butz and van Ooyen, 2013) that homeostatic structural plasticity, without any additional forms of Hebbian plasticity, can account for the changes observed in the visual cortex after focal retinal lesions: an increased dendritic spine turnover in the center of the LPZ (Keck et al., 2008), an overshoot in axonal sprouting from the peri-LPZ into the LPZ (Yamahachi et al., 2009), and a functional retinotopic remapping (Giannikopoulos and Eysel, 2006; Keck et al., 2008). In MSP, changes in topology arising from structural plasticity do not require any goal-directed network process but emerge solely from a local neuronal mechanism aimed at restoring neuronal firing rates.

Here, we investigated how local changes in connectivity brought about by homeostatic structural plasticity altered intra-area connectivity. Currently, there are no experimental studies available on intra-area topology changes after brain damage or deafferentation, but we found remarkable similarities between our model results and observed changes in inter-area connectivity especially after subcortical stroke. As a direct result of network rewiring after focal deafferentation, the model network as a whole first increased its small-worldness and then became more random and consequently less small-world. At the same time that the whole network became more random, the deafferentated neurons themselves increased their betweenness centrality if network repair was succesful. The increase in betweenness centrality may therefore hold as a biomarker for brain repair after deafferentation. The decrease in small-worldness of the whole network was associated with a decrease in local but an increase in global efficiency of the deafferented neurons, with efficiency defined as the average inverse of shortest paths between neurons. Our modeling results strongly resemble experimental and clinical data showing that during the course of post-stroke reorganization, inter-regional networks become more random, while areas that lost input as a consequence of the infarct increase their betweenness centrality (Wang et al., 2010). Thus, our model of homeostatic structural plasticity, even though at first interpretation a model for intra-area reorganization, may provide valuable insights into the mechanisms underlying inter-area topology changes during brain repair.

## 2. Materials and methods

### 2.1. The model at a glance

Our Model of Structural Plasticity (MSP) (Butz and van Ooyen, 2013; Butz et al., 2014) represents synapses not merely as synaptic weight factors but as composed of two complementary synaptic elements: an axonal element representing axonal boutons or terminals, and a dendritic element representing any postsynaptic specialization on the dendrite (e.g., a dendritic spine). Synaptic elements develop independently of their matching element in an activity-dependent manner. A neuron creates new synaptic elements when its level of electrical activity is below a homeostatic set-point and decreases the number of elements when its activity exceeds this set-point. In addition, neurons need a minimum level of activity to form synaptic elements. Newly formed elements are vacant and available for synapse formation. Vacant axonal and dendritic elements can connect to form a new synapse. Synaptic elements of adjacent neurons are more likely to connect than those of more distant neurons. Vacant synaptic elements that are not used for synapse formation decay spontaneously with a certain rate. Existing synapses can break up if an element bound in a synapse is removed by the hosting neuron. The complementary synaptic element of the broken-up synapse becomes vacant and available for synapse formation again, which enables structural rewiring of neuronal networks. The algorithm proceeds in three steps. First, electrical activity is computed for every neuron. Second, numbers of synaptic elements are updated depending on the current average level of electrical activity of each neuron, which may cause the breaking of synapses. Third, vacant synaptic elements are recombined to form new synapses. Changes in electrical activity and number of synaptic elements proceed on a continuous timescale, whereas the breaking and formation of synapses take place at discrete time steps.

### 2.2. Neuron model

The same network and neuron model was used as in Butz and van Ooyen (2013), with *n^ex^* = 320 excitatory and *n^in^* = 80 inhibitory Izhikevich neurons (Izhikevich, 2003). Inhibitory neurons only differ from excitatory ones in the sign of synaptic transmission. Excitatory neurons were placed with some jitter on a 20 x 16 grid with a spatial distance between two grid points of 150 μm. Inhibitory neurons were placed evenly between the excitatory neurons. Electrical activity is modeled by two differential equations, one for the membrane potential *v* and one for a recovery variable *u* enabling re-polarization after an action potential:

(1) $$\begin{array}{l} \frac{dv}{dt}=k_{1}v^{2}+k_{2}v+k_{3}-u+I_{syn}+I_{ext}\\ \frac{du}{dt}=a(bv-u) \end{array}$$

where *v* and *u* are in mV, *t* is in ms, *k*_1_ = 0.04 mV^−1^ms^−1^, *k*_2_ = 5 ms^−1^, and *k*_3_ = 140 mVms^−1^. Every time a neuron fires (*v* ≥ 30 mV), *v* and *u* are reset:

(2) $$\text{if }v\geq 30\text{ mV},\text{then }\left\{ \begin{array}{l} v\leftarrow c\\ u\leftarrow \;u+d \end{array}\right.$$

where *a* = 0.1 ms^−1^, *b* = 0.2 ms^−1^, *c* = −65 mV, and *d* = 2 mVms^−1^. Synaptic input *I_syn_* has a fixed strength of 1 mVms^−1^ for every synapse. Synaptic input arriving at the postsynaptic neuron is low-pass filtered by an exponential filter function $h(t)=exp(-\frac{t}{\mu })$ with decay constant μ = 5 ms. External input *I_ext_* is permanently delivered as white noise with mean 5 mVms^−1^ and standard deviation 1 mVms^−1^ according to Izhikevich (2003); Butz and van Ooyen (2013).

Intracellular calcium concentration is used as a low-passed filtered average of the firing frequency of each neuron (Butz and van Ooyen, 2013). Every time a neuron fires, calcium concentration is increased by β = 0.001 ms^−1^ and then decreases exponentially to zero with decay time τ_*Ca*_ = 10000 ms.

### 2.3. Model of structural plasticity

We used our model of structural plasticity (MSP), which is described in detail in Butz and van Ooyen (2013); Butz et al. (2014). The model proceeds in three steps: (1) updating electrical activity, as described above; (2) updating the number of synaptic elements and eventually the breaking of synapses if synaptic elements were deleted; and (3) the formation of new synapses.

#### 2.3.1. Update of synaptic elements and breaking of synapses

**Figure 1:**
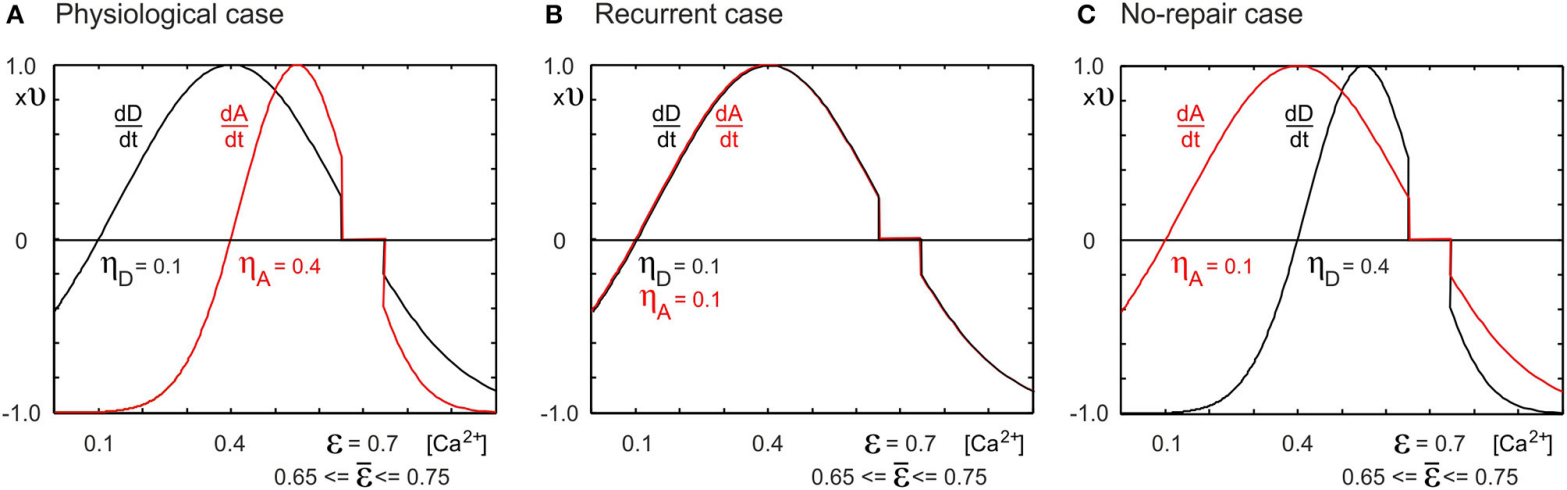
**Depending on the neuronal growth curves for the change *dD/dt* in number of dendritic elements and the change *dA/dt* in number of axonal elements, network reorganization after lesions leads to different network topologies**. Changes in the number of elements are dependent on the time-averaged neuronal electrical activity as measured by the cell's intracellular calcium concentration [*Ca*^2+^]. **(A)** If the minimal activity for dendritic element formation is lower than that for axonal element formation (η_*D*_ = 0.1, η_A_ = 0.4, respectively), networks reorganize in a physiological manner, with axonal and dendritic element dynamics (Butz and van Ooyen, 2013) resembling experimental observations (Keck et al., 2008). **(B)** If dendritic and axonal elements can already grow at low activity levels (η_*D*_ = η_A_ = 0.1), we obtain strongly recurrently connected networks after a lesion. **(C)** If dendritic elements need high levels of activity (η_*D*_ = 0.4, η_A_ = 0.1), no network repair takes place, i.e., no restoration of activity levels. We replaced the homeostatic set-point ϵ = 0.7 by a homeostatic range of 0.65 ≤ ϵ ≤ 0.75, in which no change in number of axonal or dendritic elements takes place. We chose ν = 10^−4^ ms^−1^.

We applied Gaussian growth curves (Figure 1) for the number *A_i_* of axonal elements, the number *D^ex^_i_* of excitatory dendritic elements and the number *D^in^_i_* of inhibitory dendritic elements:

(3) $$\begin{array}{l} \frac{dz_{i}}{dt}=\nu \;\left(2e^{-{\left(\frac{{\left[Ca^{2+}\right]}_{i}-\xi_{z}}{\zeta_{z}}\right)}^{2}}-1\right)\\ \;\xi_{z}=\frac{\eta_{z}+\epsilon }{2}\\ \;\zeta_{z}=\frac{\eta_{z}-\epsilon }{2\sqrt{-ln(1/2)}} \end{array}$$

where ν is the growth rate and ϵ is the homeostatic set-point, at which *dz/dt* = 0. The variable *z* needs to be replaced by the respective type of synaptic element *A*, *D^ex^*, or *D^in^*. If the calcium concentration [*Ca*^2+^]*_i_* (a measure for the average electrical activity of the neuron) is higher than ϵ, synaptic elements are removed; if it is lower than that, synaptic elements are formed. However, there is also a minimum calcium concentration required for the formation of elements: η_*A*_ for axonal elements and η_*D*_ for dendritic elements. If the concentration is lower than η_*A*_, axonal elements are removed; if it is lower than η_*D*_, dendritic elements are removed. The center and width of the Gaussian-shaped growth curve are given by ξ and ζ, respectively.

***2.3.1.1. Parameters of activity-dependent changes in synaptic elements***. For all types of elements, we chose ν = 10^−4^ ms^−1^. As in Butz and van Ooyen (2013), we studied three cases with different sets of growth curves (Figure 1): (1) η_*A*_ = 0.4, η_*D*_ = 0.1, ϵ = 0.7; (2) η_*A*_ = η_D_ = 0.1, ϵ = 0.7; and (3) η_*A*_ = 0.1, η_*D*_ = 0.4, ϵ = 0.7. The first case is referred to as the physiological case because it best reproduces experimental findings on dendritic spine and axonal bouton dynamics in the primary visual cortex after focal retinal lesion (Butz and van Ooyen, 2013). The other two cases are aberrant cases. The second case is called the recurrent case because network repair is brought about by massive recurrent connections in the LPZ. The third case is called the no-repair case because with this choice of growth parameters, neurons are not able to restore their electrical activity back to the homeostatic set-point.

Since with discrete synaptic elements there is no solution where all neurons are exactly at the homeostatic set-point, neurons will continue to rewire their connectivity at a low rate. To stop network rewiring when neurons are close to the homeostatic set-point ϵ, we replaced the set-point by a homeostatic range ϵ = [0.65..0.75]. In this range, neurons do not initiate activity-dependent changes in number of synaptic elements; i.e., *dz/dt* = 0 if 0.65 ≤ [*Ca*^2+^] ≤ 0.75.

In addition to activity-dependent changes in synaptic elements, vacant synaptic elements decay spontaneously with a very slow time constant of τ_*vac*_ = 10 updates in connectivity.

***2.3.1.2. Breaking of synapses***. Since network connectivity is updated at discrete time steps but synaptic elements change continuously over time due to the activity-dependent growth rules, it can happen that a neuron has more outgoing synapses than axonal elements or more incoming synapses than dendritic elements at the time of the next update in network connectivity. In that case, the neuron has to delete the surplus of synapses and to update connectivity.

To update connectivity, the algorithm needs to select which synapses are to be removed. All synapses have an equal chance of being deleted. Note, however, that multiple synapses can co-exist from neuron *j* to *i* and that the more synapses there are, the higher the chance that a synapse between neuron *j* and *i* will be deleted. The probability *P^del^_i,j_* for synapse deletion between neuron *j* and *i* is computed by the following master equation that captures four different cases:

(4) $$P_{i,j}^{del}=\frac{W_{i,j}}{\sum W_{k,l}}$$

For deletion of incoming synapses, we need to distinguish between excitatory and inhibitory synapses in Equation 4. For deletion of incoming excitatory synapses of neuron *i* ∈ {*In* ∪ *Ex*}, we sum up *W_k,l_* over all *l* ∈ {*Ex*}. For deletion of incoming inhibitory synapses of neuron *i* ∈ {*In* ∪ *Ex*}, we sum up *W_k,l_* over all *l* ∈ {*In*}. For deletion of outgoing excitatory synapses of excitatory presynaptic neuron *j* ∈ {*Ex*}, all synapses are considered to any postsynaptic neuron *k* ∈ {*In* ∪ *Ex*}. Thus, we sum up *W_k,l_* over all *k* ∈ {*In* ∪ *Ex*}. The same holds true for outgoing inhibitory synapses with *j* ∈ {*In*}.

Sequentially, outgoing and incoming excitatory and inhibitory synapses were selected for deletion. For every type of synapse, the accumulated sum of *P^del^_i,j_* (see description of Equation 4 for the range of *i* and *j*) gave a probability distribution from which we drew the required number of synapses to be deleted. The selected synapse was deleted by reducing the respective entry *W_i,j_* in the connectivity matrix by one. It can happen that more than one synapse is selected for deletion from the same connection *j* to *i*. In that case, the implementation of the algorithm made sure that the number of synapses to be deleted did not exceed *W_i,j_*. Whenever a neuron deletes a synaptic element that is bound in a synapse, the complementary synaptic element on the other neuron remains and becomes vacant again.

#### 2.3.2. Synapse formation

For synapse formation, the algorithm checked whether a neuron gained vacant synaptic elements, i.e., whether the total number of synaptic elements exceeded the number of bound synaptic elements of this type. Matching vacant synaptic elements (vacant excitatory axonal elements *A^vac^_j_*, *j* ∈ {*Ex*}, with vacant excitatory dendritic elements *D^ex,vac^_i_*, and vacant inhibitory axonal elements *A^vac^_j_*, *j* ∈ {*In*}, with vacant inhibitory dendritic elements *D^in,vac^_i_*) were randomly connected among each other with probability density function *P^form^*. The probability *P^form^_i,j_* for forming new synapses between neuron *j* and *i* depended on the number of vacant synaptic elements they offered and on the Euclidean distance between neuron *j* and *i*:

(5) $$\begin{array}{l} P_{i,j}^{form}=\left\{\begin{array}{l} j\in \{Ex\}\;:\;\frac{A_{j}^{vac}\;D_{i}^{ex,vac}}{\sum_{\iota \in \{Ex\}}A_{\iota }^{vac}\;\sum_{\kappa \in \{Ex\cup In\}}D_{\kappa }^{ex,vac}}\;K_{ij}\\ j\in \{In\}\;:\;\frac{A_{j}^{vac}\;D_{i}^{in,vac}}{\sum_{\iota \in \{In\}}A_{\iota }^{vac}\;\sum_{\kappa \in \{Ex\cup In\}}D_{\kappa }^{in,vac}}\;K_{ij} \end{array}\right\}\\ \text{ with }i\in \{Ex\cup In\}. \end{array}$$

where *K_i,j_* is the Euclidean distance-dependent likelihood (kernel function) that neuron *j* connects to neuron *i* at all, irrespective of the number of vacant elements *i* and *j* offer. As in our previous work on MSP (Butz and van Ooyen, 2013; Butz et al., 2014), we applied either a flat kernel *K_i,j_* = 1 (creating random networks) or a two-dimensional Gaussian kernel (creating small-world networks):

(6) $$K_{i,j,\;i\neq j}=e^{-\frac{{\left(pos_{xj}-pos_{xi}\right)}^{2}+{\left(pos_{yj}-pos_{yi}\right)}^{2}}{\sigma^{2}}}$$

with *pos_xi_* the x-coordinate and *pos_yi_* the y-coordinate of postsynaptic neuron *i*, and *pos_xj_* and *pos_yj_* the coordinates of presynaptic neuron *j*. The probability for autapse connections (i.e., a neuron connecting to itself) was set to zero (*K_i,j_* = 0 for *i* = *j*). For these simulations, we chose σ = 1 × 150 μm, where 150 μm is the distance between two grid points. Because *K* only depends on the Euclidean distance between neurons and since neurons do not migrate, *K* remains fixed.

For every update in connectivity, the minor number of vacant excitatory and inhibitory axonal or dendritic elements determined how many new excitatory and inhibitory synapses, respectively, could at most be formed (so-called potential synapses). Thus, the number of excitatory and inhibitory potential synapses equaled

(7) $$\begin{array}{l} M^{PotSyn,ex}=\min\left(\sum_{\iota \in \{Ex\}}A_{\iota }^{vac},\;\sum_{\kappa \in \{Ex\cup In\}}D_{\kappa }^{ex,vac}\right)\\ M^{PotSyn,in}=\min\left(\sum_{\iota \in \{In\}}\;A_{\iota }^{vac},\;\sum_{\kappa \in \{Ex\cup In\}}D_{\kappa }^{in,vac}\right) \end{array}$$

for every update in connectivity.

From this distribution, the algorithm chose at maximum *M^PotSyn,ex^* excitatory and *M^PotSyn,in^* inhibitory connections at which new synapses were created. The respective entries *W_i,j_* in the connectivity matrix were then increased by one. A connection was chosen by drawing a random number from a uniform distribution and comparing it to the accumulated probabilities *P^form^_i,j_* for all excitatory connections and all inhibitory connections of the entire network. That connection was chosen that had the highest accumulated probability that the random number just did not exceed. If, for this try, the random number exceeded all accumulated probabilities, no synapse was formed. Hence, not necessarily all of the potential synapses were formed.

Additionally, synapse formation needed to fulfill the condition that the number *W*^+^_*i,j*_ of newly formed synapses from neuron *j* to *i* did not exceed the number of vacant synaptic elements that neuron *j* and *i* offered:

(8) $$\begin{array}{l} W_{i,j}^{+}\leq \;\left\{\begin{array}{l} j\in \{Ex\}\;:\;\min(A_{j}^{vac}\;,D_{i}^{ex,vac})\\ j\in \{In\}\;:\;\min(A_{j}^{vac}\;,D_{i}^{in,vac}) \end{array}\right\}\\ \text{ with }i\in \{Ex\cup In\}. \end{array}$$

In every update, this condition was checked and synapse formation infringing this condition was rejected. Alternatively, update of connectivity can also be implemented in a purely local fashion (Butz and van Ooyen, 2013).

### 2.4. Modeling deafferentation

We grew every model network from scratch, i.e., starting with zero connectivity and zero synaptic elements. Networks were formed by exactly the same growth rules that were effective after the lesion. However, in order to grow networks from scratch, it was necessary to use initially a higher level of external input. We used *I_ext_* = 8 mVms^−1^ for the first 500 updates in connectivity and then lowered it gradually down to 5 mVms^−1^ according to *I_ext_(T)* = ((8−5)/(1+*exp((T*−500)/200))+5) mVms^−1^. At *T* = 8000, we removed the input of a circumscribed area, the lesion projection zone (LPZ), by setting *I_ext,LPZ_(T)* = 0 (for *T* ≥ 8000) permanently. The LPZ spans from *x*1 = 5× 150 μm to *x*2 = 12× 150 μm and from *y*1 = 5× 150 μm to *y*2 = 12× 150 μm (cf. **Figure 5**) for all simulations and all cases (cf. Update of synaptic elements and breaking of synapses). We refer to the rest of the network with intact input as “intact zone.” Every simulation is continued for another *T* = 12000 updates in connectivity. As in our previous work (Butz and van Ooyen, 2013), we matched 1000 updates in connectivity with 14 days post-lesion. Thus, simulations predict the time course of network rewiring for 24 weeks after the lesion.

### 2.5. Topology measurements

A neuronal network can be seen as a graph, with neurons as nodes and synapses as edges or links between nodes. Since the presynaptic neuron always activates the postsynaptic neuron (and never the other way round), we regard the graph as directed. In order to describe changes in network topology after a focal loss of input, we assessed the following graph theoretical measures at every update in connectivity. To reduce the complexity of the assessment, we considered only the topology of the excitatory synaptic connections *W^ex,ex^* between the *n^ex^* excitatory neurons. For the graph theoretical assessments, the brain connectivity toolbox by Rubinov and Sporns was used (Rubinov and Sporns, 2010).

#### 2.5.1. Weighted characteristic path length

The characteristic path length *L* measures the average shortest path from one (excitatory) neuron to any other (excitatory) neuron in the network. Path length is defined as the number of connections that needs to be traveled to go from one neuron (possibly via intermediate neurons) to any other neuron:

(9) $$L=\frac{1}{n^{ex}}\sum_{i}^{n^{ex}}L_{i}=\frac{1}{n^{ex}}\sum_{i}^{n^{ex}}\frac{\sum_{j,j\neq i}^{n^{ex}}d_{ij}}{n^{ex}-1}$$

On top of this definition, a direct connection between two neurons in a weighted network is considered “shorter” the stronger the weight of the connection is. For our network model, we take the number of synapses *W^ex,ex^_i,j_* between two directly linked neurons *j* and *i*, with *i,j* ∈ {*Ex*}, as the weight of the connection and the inverse 1/*W^ex,ex^_i,j_* as the length *l_i,j_* of the connection. The shortest path *d_i,j_* is then the smallest sum of connection lengths that lead from neuron *j* to *i* via any intermediate neurons. We calculated the weighted characteristic path length according to Rubinov and Sporns (2010). Additionally, in order to study the connectivity between subnetworks, we used Equation 9 to compute the average path lengths from neurons in the intact zone (with intact input) to neurons in the LPZ (deprived of input) and vice versa.

#### 2.5.2. Weighted clustering coefficient

The clustering coefficient is an indication for how strongly neurons in a network are interconnected. It measures how many of any two neurons *j* and *h* that are both connected to node *i* are also connected to each other, relative to all neurons connected to *i*:

(10) $$C=\frac{1}{n^{ex}}\sum_{i}^{n^{ex}}C_{i}=\frac{1}{n^{ex}}\sum_{i}^{n^{ex}}\frac{\sum_{j,h}^{n^{ex}}a_{ij}a_{ih}a_{jh}}{k_{i}(k_{i}-1)}$$

where *a_ij_, a_ih_, a_jh_* ∈ {0, 1} (1 if a connection between the respective neurons exists and 0 if not) and *k_i_* is the number of neurons that neuron *i* is connected to. For weighted directed networks, the clustering coefficient can be computed according to the formalism by Fagiolo (2007). We computed the clustering coefficient at every update in connectivity according to the implementation by Rubinov and Sporns (2010). In addition to the averaged clustering coefficient of the entire network, we also computed the clustering coefficient averaged over either the LPZ neurons only or over the intact zone neurons only.

#### 2.5.3. Small-world parameter

To estimate the small-worldness of networks, we applied the formalism by Humphries and Gurney (2008):

(11) $$S=\frac{\gamma }{\lambda }=\frac{C/C^{rand}}{L/L^{rand}}$$

We replaced the clustering coefficient *C* and the characteristic path length *L* by the version for weighted directed graphs as described above. To obtain the normalized clustering coefficient γ and the normalized characteristic path length λ, *C* and *L* were divided by *C^rand^* and *L^rand^*, respectively, taken from an Erdős-Rényi random graph generated with the same number of neurons and synapses as in the deafferentated networks at every update in connectivity.

#### 2.5.4. Betweenness centrality

Betweenness centrality measures the importance of neurons in the network. Betweenness centrality of a neuron is calculated by summing up the number of all shortest paths in the network that go via this neuron and dividing it by the number of all other shortest paths that do not pass this neuron. Global betweenness centrality is the sum over the betweenness centrality of all neurons:

(12) $$BC_{global}=\sum_{i}^{n^{ex}}\sum_{k\neq i\neq l}\frac{\sigma_{kl}(i)}{\sigma_{kl}}$$

where σ_*kl*_ is the total number of multiple shortest paths between neuron *k* and neuron *l*, and σ*_kl_(i)* is the number of shortest paths that go via neuron *i*. Shortest paths are based on weighted excitatory connections *W^ex,ex^_i,j_*, and global betweenness centrality was computed by the formalism for weighted directed networks by Brandes (2001) as implemented by Rubinov and Sporns (2010).

#### 2.5.5. Local efficiency

Local efficiency *E_loc,i_* measures how well the neighbors of neuron *i*, i.e., other neurons that directly form a synapse with *i*, are interconnected and is therefore related to the clustering coefficient. For this, the average of the shortest path lengths *d_jh_(G^ex^_i_)* between any two excitatory neighboring neurons *j* and *h* of neuron *i* is computed that uses only paths of the subgraph *G^ex^_i_* consisting of all the excitatory neighbors of *i* but not of *i* itself (Latora and Marchiori, 2001):

(13) $$\begin{array}{l} E_{loc}=\frac{1}{n^{ex}}\sum_{i}^{n^{ex}}E_{loc,i}\\ \;=\frac{1}{n^{ex}}\sum_{i}^{n^{ex}}\frac{\sum_{j,h,\;j,h\neq i}^{n^{ex}}a_{ij}a_{jh}{\left[d_{jh}(G_{i}^{ex})\right]}^{-1}}{k_{i}(k_{i}-1)} \end{array}$$

where *a_ij_, a_jh_* ∈ {0, 1} (1 if a connection between the respective neurons exists and 0 if not) and *k_i_* is the number of neurons that neuron *i* is connected to. We used the weighted, directed version of local efficiency (Rubinov and Sporns, 2010).

#### 2.5.6. Global efficiency

Global efficiency *E_glob_* is related to the inverse of the characteristic path length, but with the advantage that it can also be meaningfully computed for unconnected graphs. Whereas the path length between unconnected nodes is infinite (cf. Equation 9), the inverse is zero and therefore adds neutrally to global efficiency (Latora and Marchiori, 2001; Achard and Bullmore, 2007):

(14) $$E_{glob}=\frac{1}{n^{ex}}\sum_{i}^{n^{ex}}E_{glob,i}=\frac{1}{n^{ex}}\sum_{i}^{n^{ex}}\frac{\sum_{j,j\neq i}^{n^{ex}}d_{ij}^{-1}}{n^{ex}-1}$$

where *E_glob,i_* is the efficiency of node *i* and *n^ex^* is the number of excitatory neurons. We used the version of this equation for weighted, directed graphs (Rubinov and Sporns, 2010). Note that local efficiency and clustering coefficient as well as global efficiency and characteristic path length are closely related but not identical measures. Local and global efficiency are frequently used in clinical studies and are therefore presented here in addition to clustering coefficient and characteristic path length.

## 3. Results

### 3.1. Physiological network rewiring

**Figure 2:**
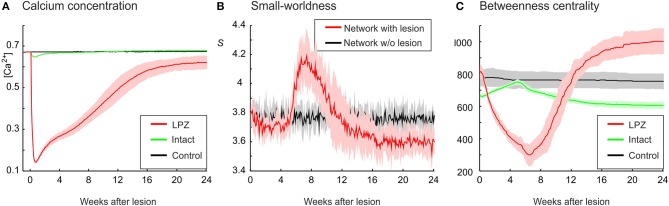
**Physiological case**. Compensatory network rewiring renders neuronal networks more random and increases their betweenness centrality. **(A)** Average electrical activities, as measured by the mean calcium concentration of the respective area, are restored to the homeostatic range for neurons in the LPZ (red) and the intact zone (green). Neurons corresponding to the LPZ in a non-lesioned network do not alter their calcium concentration (control, black). **(B)** Networks become more random after deafferentation, as indicated by a decrease in small-world parameter *S* (red) measured over the entire network, whereas control networks show no change in small-worldness (black). **(C)** At the same time, betweenness centrality increases in the LPZ (red) but decreases in the intact zone (green). Betweenness centrality of neurons corresponding to the LPZ in a non-lesioned network remains stable (control, black). Means over five simulations per scenario. Shadings of the curves indicate standard deviations.

In our previous work (Butz and van Ooyen, 2013), we postulated activity-dependent growth curves for axonal and dendritic elements that gave rise to the same kind of network rewiring as observed in primary visual cortex after focal retinal lesions. With these growth curves (referred to as physiological growth curves), in which axonal elements required higher levels of electrical activity than dendritic elements to grow out (η_*A*_ = 0.4, η_*D*_ = 0.1), the LPZ recovered from the outside to the inside and the turnover of dendritic elements was surprisingly similar to the experimental data on dendrtic spine turnover (Butz and van Ooyen, 2013). In the present study, we investigated how network topology changes in response to a focal loss of input, with neurons rewiring their inputs (and outputs) locally in order to restore a desired level of electrical activity. Our modeling results show that networks employing physiological growth curves return to a homeostatic range in electrical activity (Figure 2A) and, as a result of compensatory rewiring, become more randomly connected, as indicated by a lower value of the small-world parameter *S* (Figure 2B) measured over the entire network. Although random networks have no nodes of particular importance and hence a low betweenness centrality, neurons in the LPZ have a higher betweenness centrality after network rewiring than before the lesion (Figure 2C).

**Figure 3:**
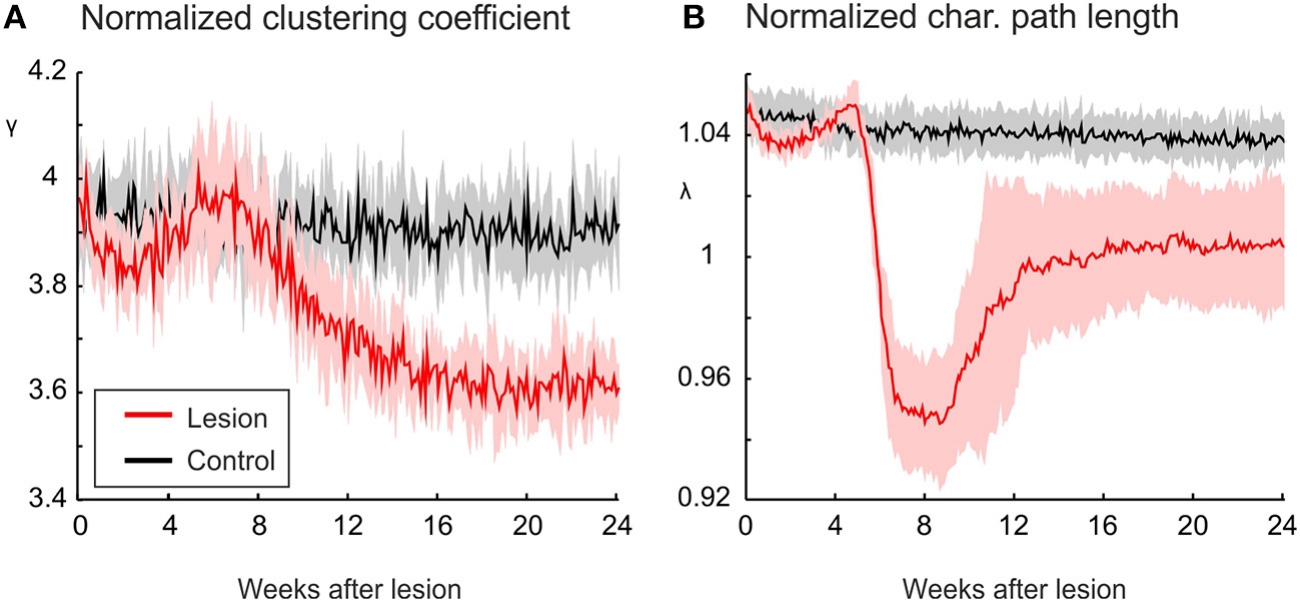
**The increasing randomness of networks after deafferentation is due to a marked decrease in clustering, as shown by a decrease in the normalized clustering coefficient γ (A)**. The average of shortest paths, as measured by the normalized characteristic path length λ (Equation 11), shows only very little change in absolute terms **(B)**. Means over five simulations per scenario. Shadings of the curves indicate standard deviations.

The decrease in small-world parameter *S* is determined by the course of the clustering coefficient γ and the characteristic path length λ. While λ converges to one, γ decreases markedly (Figure 3) and is thereby responsible for networks becoming more random. The decrease in clustering is not immediate but sets in between 6 and 8 weeks after the lesion. As will be shown below, it takes some time until network reorganization has managed to restore neuronal activities to their homeostatic range. During this time period, there is a temporary drop in characteristic path length below one, which contributes to a temporary rise in *S* (Figure 2B). However, after about 16 weeks, λ reaches stable values around one. From the same time on, *S* stabilizes at lower levels than in control networks without lesions.

**Figure 4:**
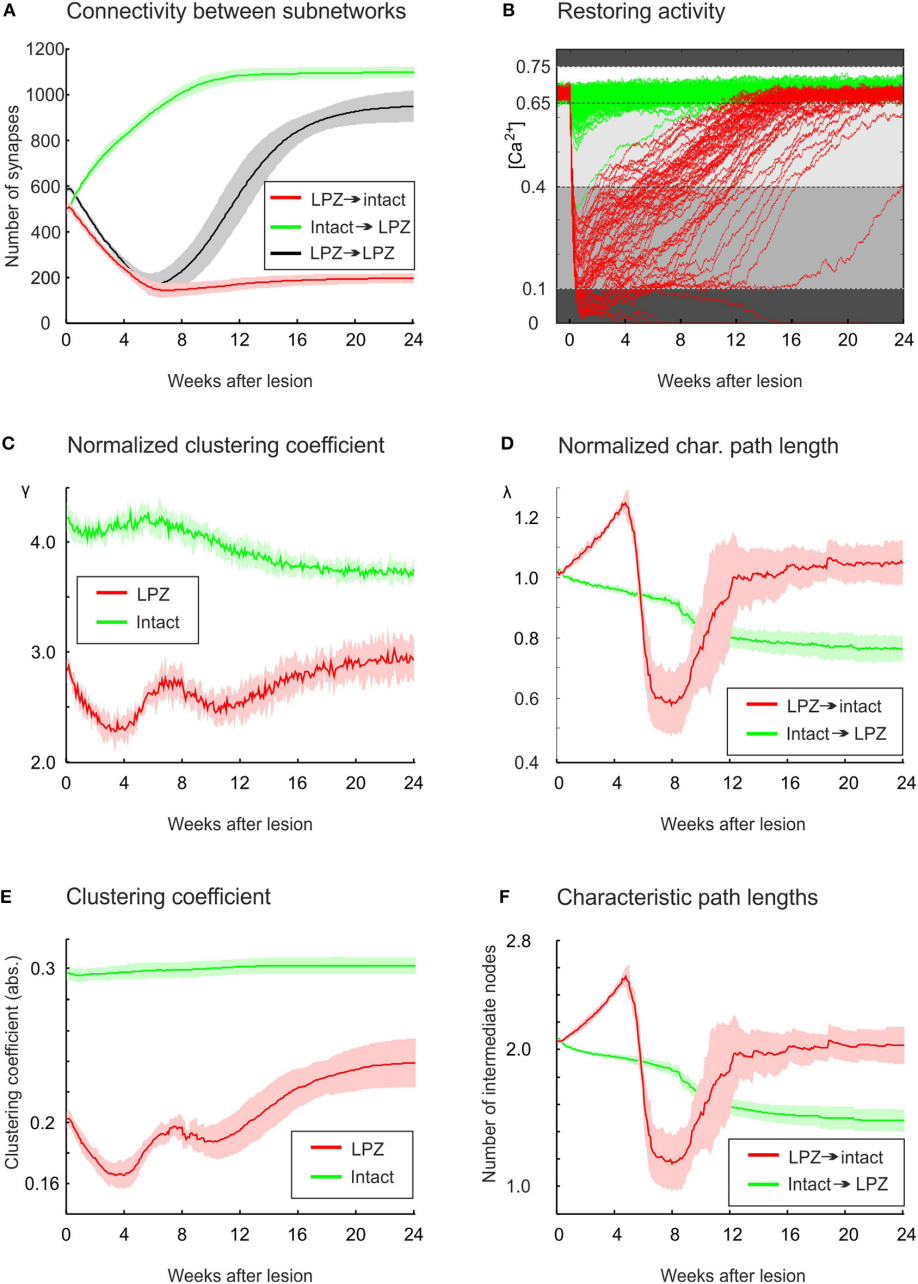
**In the physiological case, compensatory network rewiring relies on the formation of new synapses from the intact zone to the LPZ**. **(A)** Synapse numbers from the intact zone to the LPZ increase (green), while synapses numbers from the LPZ to the intact zone decrease (red). **(B)** All neurons in the intact zone (green) and most neurons in the LPZ (red) return to the homeostatic range following deafferentation. Neurons lose axonal and dendritic elements if their calcium concentration is lower than 0.1 or higher than 0.75 (dark gray background). Neurons form only dendritic elements if their calcium concentration is greater than 0.1 but lower than 0.4 (gray), and form both axonal and dendritic elements if their calcium concentration is greater 0.4 but lower than 0.65 (light gray). The homeostatic range, in which synaptic element numbers do not change, spans from 0.65 to 0.75. The diagram helps to match changes in topology with the current level of electrical activity. **(C)** The normalized average clustering coefficient γ of neurons in the LPZ (including connections with the entire network) decreases while neuronal activities are very low (<0.1) and increases as soon as activities of LPZ neurons are greater than 0.1. The first bump in clustering is brought about by ingrowing synapses from the intact zone into the LPZ, whereas the second rise in clustering is caused predominantly by new synapses within the LPZ, which are formed when calcium concentrations of LPZ neurons exceed 0.4. The γ of neurons in the intact zone (considering all their connections to any neuron in the entire network) decreases continuously after a temporary rise. **(D)** Average shortest paths from neurons in the intact zone to neurons in the LPZ show a steady decrease (green), while average path lengths from LPZ to intact zone neurons return to initial levels after a tri-phasic increase and decrease. **(E)** The clustering coefficient with no normalization (Equation 10) does not show a decrease for intact zone neurons as the normalized clustering coefficient γ does. **(F)** No differences were found between the characteristic path length and the normalized characteristic path length λ. Green curve indicates changes in clustering coefficient of intact zone neurons with the entire network. Means over five simulations per scenario. Shadings of the curves in **(A,C–F)** indicate standard deviations.

From our previous work on modeling cortical rewiring after focal retinal lesions (Butz and van Ooyen, 2013), we know that functional network repair can be brought about by an, also experimentally observed, ingrowth of connections from the intact zone to the LPZ (Darian-Smith and Gilbert, 1994; Yamahachi et al., 2009). For physiological network repair to go along with functional retinotopic remapping (as shown in mice Keck et al., 2008), we found that it is important that the majority of new connections impinging on deafferentated neurons originates from intact areas and transmits electrical activity from the intact zone to the LPZ. Here, we further investigate whether the changes in global topology parameters express this ingrowth of connections. For this, we first focus on the activity-dependent changes in synapse numbers and connectivity between the intact zone and the LPZ. The first 6 weeks are dominated by a loss of synapses originating from the LPZ (Figure 4A). This is a direct consequence of neuronal activities being low and calcium concentrations being below η_*A*_ = 0.4 (Figure 4B), which causes axonal elements to be removed. By contrast, axonal elements from the intact zone form additional synapses with the LPZ right from the onset of the lesion. Between 6 and 8 weeks after the lesion, most neurons in the LPZ have reached calcium levels of 0.4 and start forming additional axonal elements, connecting to targets in the LPZ as well as the intact zone. The number of recurrent synapses from the LPZ to the LPZ does thereby at no time exceeds the number of synapses from the intact zone to the LPZ, as required for a functional remapping to emerge.

The change in λ and γ as shown in Figure 3 is measured over the entire network. We further want to understand whether the course of γ and λ is caused by the changing connectivity between the intact zone and the LPZ. For this, we assessed γ and λ for the set of LPZ and intact zone neurons separately. We can distinguish three phases in the time course of both parameters. These phases arise from the interaction between the loss of connections from the LPZ and the formation of new connections from the intact zone. The initial phase lasts for the first 4 weeks after the lesion and is dominated by a loss of connections from the LPZ. This is reflected by a decrease in γ, especially of LPZ neurons but to a lesser extent also of intact zone neurons (Figure 4C). At the same time, λ of paths from the LPZ to the intact zone increases (Figure 4D) due to the loss of connections from the LPZ to the intact zone. Conversely, λ of paths from the intact zone to the LPZ decreases because new connections are being formed originating from the intact zone.

During the second phase, roughly between 4 and 8 week, we see a temporal increase in γ of both the LPZ and the intact zone neurons (Figure 4C). This increase essentially contributes to the temporal increase in small-worldness of repairing networks as shown in Figure 2B. During this phase, the decrease in number of connections from the LPZ slows down, while new connections from the intact zone are still being formed. During this second phase, especially λ for paths from the LPZ to the intact zone shows a rapid decrease (Figure 4D). This rapid decrease is brought about by a few new connections that are formed as soon as LPZ neurons reach calcium levels of 0.4 (Figure 4B). This happens already slightly before the average number of synapses from the LPZ to the intact zone increases significantly at about 6 weeks after lesion.

A third phase can be distinguished from 8 weeks after the lesion onwards, when LPZ neurons start forming outgoing connections again. Especially the recurrent connections inside the LPZ (Figure 4A) lead to an increase in γ of LPZ neurons (Figure 4C), while neurons in the intact zone show a decrease in γ after the temporary rise. However, γ of the LPZ neurons is not strictly increasing over time; between 8 and 12 weeks after the lesion, γ decreases a second time before it finally increases toward a stable level. We can explain this fluctuation in γ by the ongoing replacement of connections during this period. Only if all neurons in the LPZ have reached calcium levels beyond 0.4, and hence contribute to axonal element and (outgoing) synapse formation, does the clustering coefficient strictly increase until rewiring comes to a standstill. During the third phase, λ of paths from intact zone to LPZ further decreases (Figure 4D). This further decrease is brought about by additional connections inside the LPZ, contributing to network repair and shortening paths to neurons in the LPZ. The decrease in path lengths to the LPZ also explains the increasing betweenness centrality of LPZ neurons, since betweenness centrality by definition is a measure of how many shortest paths go via certain nodes. As shown in Figure 4D, λ of paths from the LPZ to the intact zone takes on values of a randomized network.

Interestingly, the absolute clustering of neurons in the intact zone shows very little change (Figure 4E), implicating that the particular course of γ arises from changes in the number of connections and their clustering in comparison with a randomized network. By contrast, the changes in clustering of the LPZ (Figure 4E) as well as the characteristic path length for both the LPZ and the intact zone (Figure 4F) show similar courses for the non-normalized and normalized values. Therefore, we may conclude that networks become more random because of the increase in number of connections, whereas the increase in betweenness centrality (as a result of decreasing path lengths from the intact zone to the LPZ) is a consequence of added specific projections from the intact zone to the LPZ.

### 3.2. Aberrant network rewiring

Network repair does not in all cases lead to the formation of synapses from the outside to the inside and a functional reorganization of connectivity. In our previous study (Butz and van Ooyen, 2013), we identified three different cases of network rewiring depending on the relative values of the growth parameters η_*A*_ and η_*D*_. For η_*A*_ > η_*D*_, we observed network repair in line with the exeperimental data (physiological case); for η_*A*_ = η_D_ = 0.1, we observed network repair brought about by massive recurrent connections (recurrent case); and for η_*D*_ > η_*A*_, we observed no network repair at all (no-repair case).

**Figure 5:**
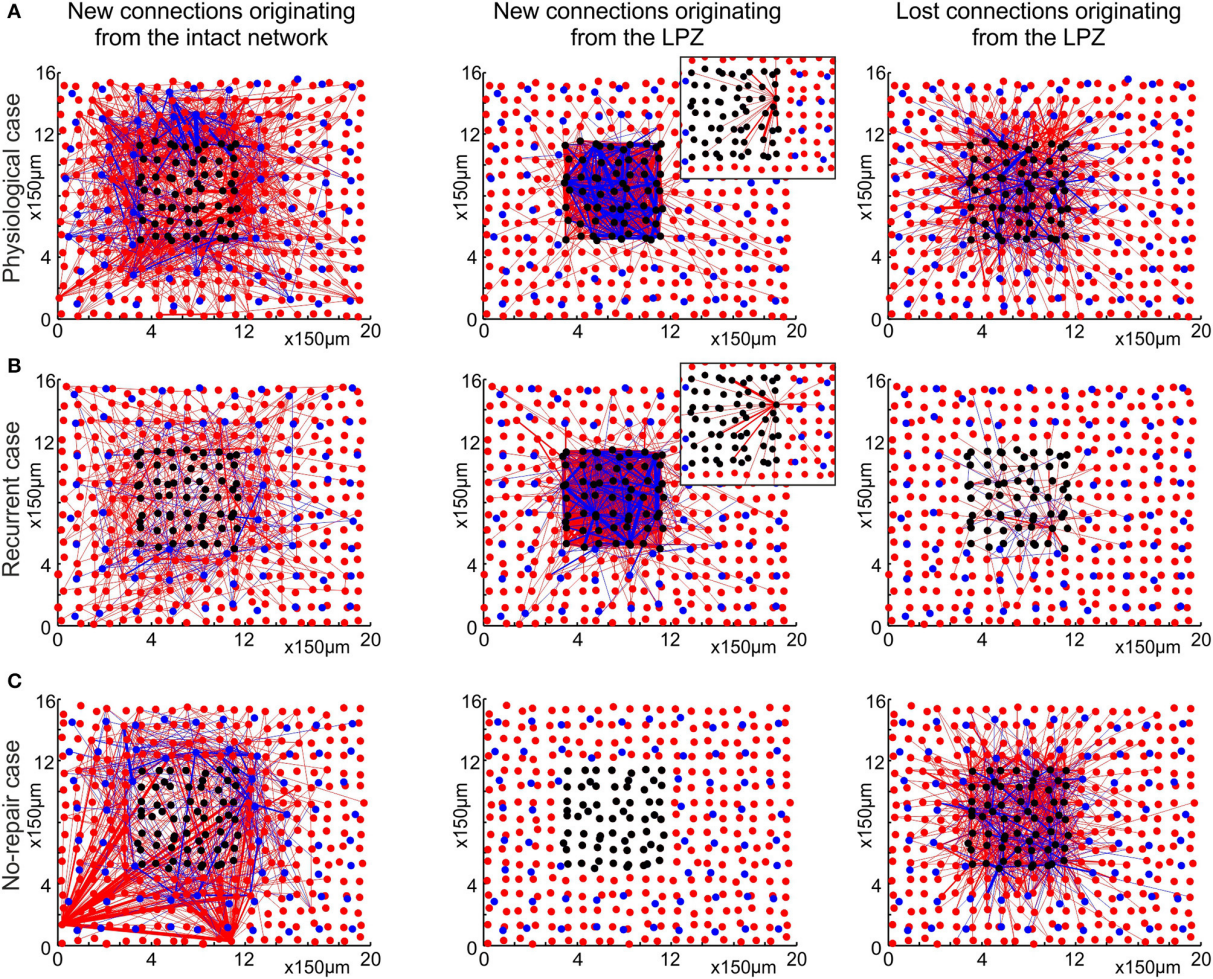
**The physiological case (A) is characterized by a pronounced replacement of synapses, whereas the recurrent case (B) predominantly adds new synapses and keeps pre-existing ones**. The no-repair case **(C)** does not form sufficient additional synapses to the LPZ. The figures show the two-dimensional layout of the network, with excitatory neurons (red dots), excitatory synaptic connections (red lines), inhibitory neurons (blue dots) and inhibitory synaptic connections (blue lines). Black dots indicate deafferentated neurons. The left column shows new synapses originating from anywhere in the intact zone. Whereas the preferred target of new synapses in the physiological case is the LPZ, only few new synapses from the intact zone to the LPZ are formed in the recurrent case. Middle column shows that most of the new synapses originating from the LPZ terminate in the LPZ in both the physiological and the recurrent case. Insets in the middle column illustrate the axonal projection pattern of an individual neuron in the LPZ. In the physiological case, neurons at the border of the LPZ connect to neurons more central in the LPZ, whereas in the recurrent case neurons have less preferrence for particular targets. The right column shows that many synapses originating from the LPZ are deleted in the physiological case but not in the recurrent case. All measurements are based on the difference between the number of synapses present before (*T*_0_ = 7950) and after the lesion (*T*_1_ = 20000 updates in connectivity, corresponding to 24 weeks after lesion), separately for excitatory and inhibitory synapses. Only excitatory neurons and excitatory to excitatory connections were used in the topological assessments.

The network rewiring occurring in the last two cases are referred to as aberrant network rewiring. Figure 5 depicts the most evident differences in the layout of connections after compensatory network rewiring between the physiological and the recurrent case and shows the no-repair case for the sake of completeness.

**Figure 6:**
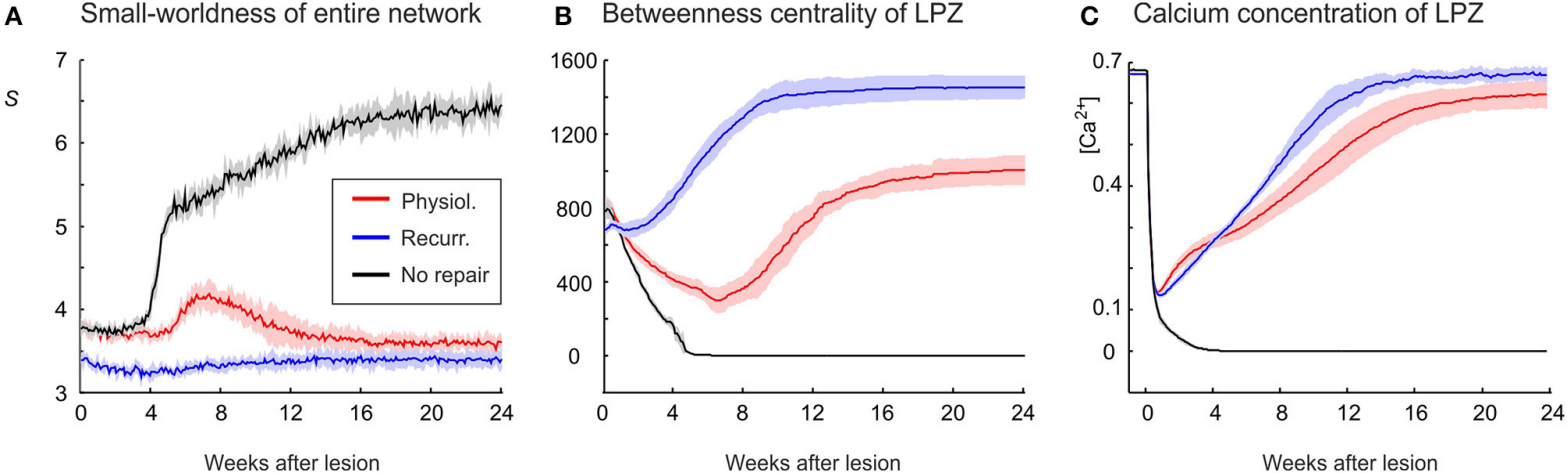
**An increasing randomness of the whole network in association with an increasing betweenness centrality of LPZ neurons after deafferentation emerges only in the physiological case. (A)** Whereas the no-repair case (black) shows an increase in small-world parameter *S* and the recurrent case (blue) shows little change in *S*, the physiological case shows a clear decrease in *S* (red). **(B)** The recurrent case shows the strongest increase in betweenness centrality. **(C)** The average calcium concentrations of the LPZ and intact zone return quickest to the homeostatic range in the recurrent case. Means over five simulations per scenario. Shadings of the curves indicate standard deviations.

Whereas in the physiological case (Figure 5A) most of the newly formed synapses from the intact zone terminate in the LPZ, we do not see this ingrowing of new synapses in the recurrent case (Figure 5B) or in the no-repair case (Figure 5C). In the recurrent case and the no-repair case, new synapses from anywhere in the intact zone predominantly connect to neurons in the intact zone in the direct vicinity of the LPZ. In the pysiological and the recurrent case, but not in the no-repair case, LPZ neurons contribute to network repair by forming additional synapses. However, there is an important difference between the physiological and the recurrent case in where LPZ neurons project to. LPZ neurons in the physiological case form new connections to neurons in the LPZ and preferentially to those in its center (inset Figure 5A), whereas LPZ neurons in the recurrent case also project to neurons in the intact zone and show less projection preference (inset Figure 5B). A marked difference between the physiological and the recurrent case is seen in the loss of synapses originating from the LPZ. Whereas many synapses are lost in the physiological case, almost no synapses originating from the LPZ are eliminated in the recurrent case. Therefore, network repair in the recurrent case is brought about by addition of new synapses, whereas in the physiological case network repair goes along with a replacement of synapses. The no-repair case shows a considerable loss of synapses originating from the LPZ. Neurons in the LPZ are not able to raise their activity beyond η_*A*_ (Figure 6C) and therefore lose axonal elements and outgoing synapses as a direct consequence of the growth rules.

Whereas physiological network repair goes along with increasing randomness of network connectivity, as indicated by a decrease in small-world parameter *S*, we do not see a considerable change in *S* in the recurrent case (Figure 6A). Interestingly, in networks that lack repair after lesions, we even see an increase in *S*. The strongest increase in betweenness centrality of neurons in the LPZ is observed in the recurrent case (Figure 6B) and sets in much earlier (after about 2 weeks) than in the physiological case due to a mere addition of synapses rather than a replacement of synapses. Betweenness centrality goes to zero (Figure 6B) when neurons do not return to the homeostatic range in activity (Figure 6C). Given that in the recurrent case, neurons in the LPZ restore their activity most quickly and completely and with the strongest increase in betweenness centrality, we may conclude that the increase in betweenness centrality is an indicator for the success of network repair in terms of restoring neuronal activity.

**Figure 7:**
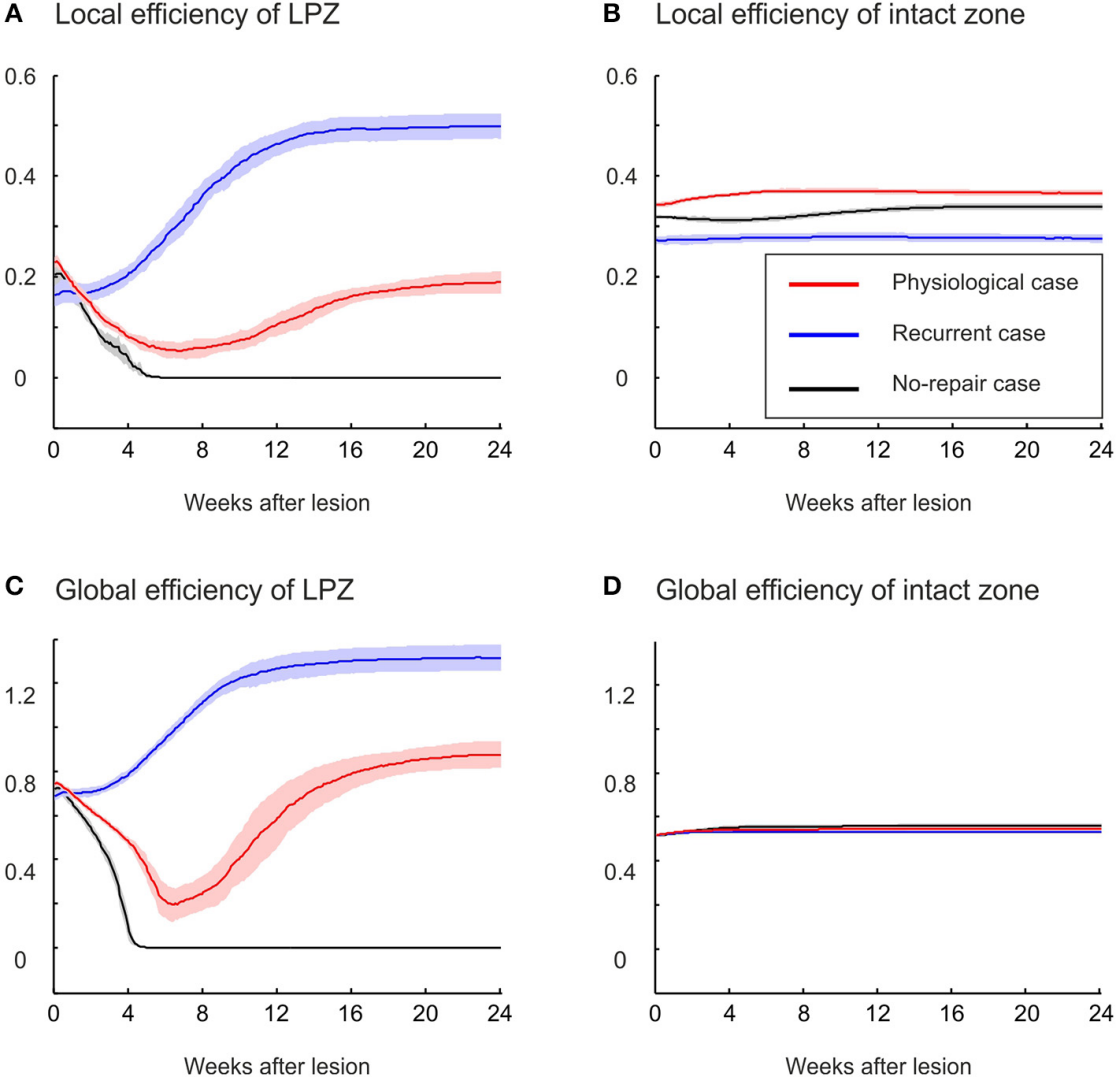
**Local and global efficiencies of LPZ neurons, but not of intact zone neurons, change as a consequence of network rewiring. (A)** The local efficiency of neurons in the LPZ increases strongest after deafferentation in the recurrent case. In the physiological case, local efficiency of LPZ neurons first decreases and later increases but remains lower than its initial value. If networks do not recover, local efficiency decreases and remains low. **(C)** Global efficiency increases in the physiological case (after a transient decrease) and in the recurrent case (without a transient decrease). The no-repair case shows a decrease in global efficiency. No changes were observed in either local **(B)** or global efficiency of neurons in the intact zone **(D)**.

Local and global efficiency are additional measures quantifying changes in network topology (Equations 13 and 14). Global efficiency indicates how efficiently information can travel through the entire network; i.e., global efficiency is the averaged sum of the inverse of the shortest paths between any two neurons in the entire network. By contrast, local efficiency of neuron *i* measures how efficiently information can be exchanged among neurons that are connected to neuron *i*; i.e., local efficiency is the averaged sum of the inverse of the shortest path between any two neurons connected to neuron *i* (excluding neuron *i* itself). Especially in sparsely connected networks, efficiency as a topology measure is preferred over characteristic path length and clustering coefficient because it can be meaningfully computed also for unconnected neurons. In the physiological case, we observe a decrease in local efficiency (Figure 7A) but an increase in global efficiency (Figure 7C) relative to the efficiencies before the lesion. Both local and global efficiency go through an initial phase in which they decrease, reaching a minimum at about 6 weeks after the lesion. The global efficiency recovers and finally even exceeds its initial level, whereas the local efficiency recovers little and remains lower than before the lesion. By contrast, in the recurrent case, both local and global efficiency increase immediately after the lesion and exceed by far their initial levels and the levels in the physiological case. A drop in local and global efficiency is observed when no network repair takes place. The intact zone does not show a considerable change in either local or global efficiency (Figures 7B,D). The ratio of local to global efficiency indicates the relative amount of local clustered and global long-range connectivity. The stronger increase in global than in local efficiency in the physiological case reflects the increasing ramdomness (cf. Figure 2B), whereas recurrent networks with a strong increase in global and local efficiency become even more small-world (cf. Figure 6A).

**Figure 8:**
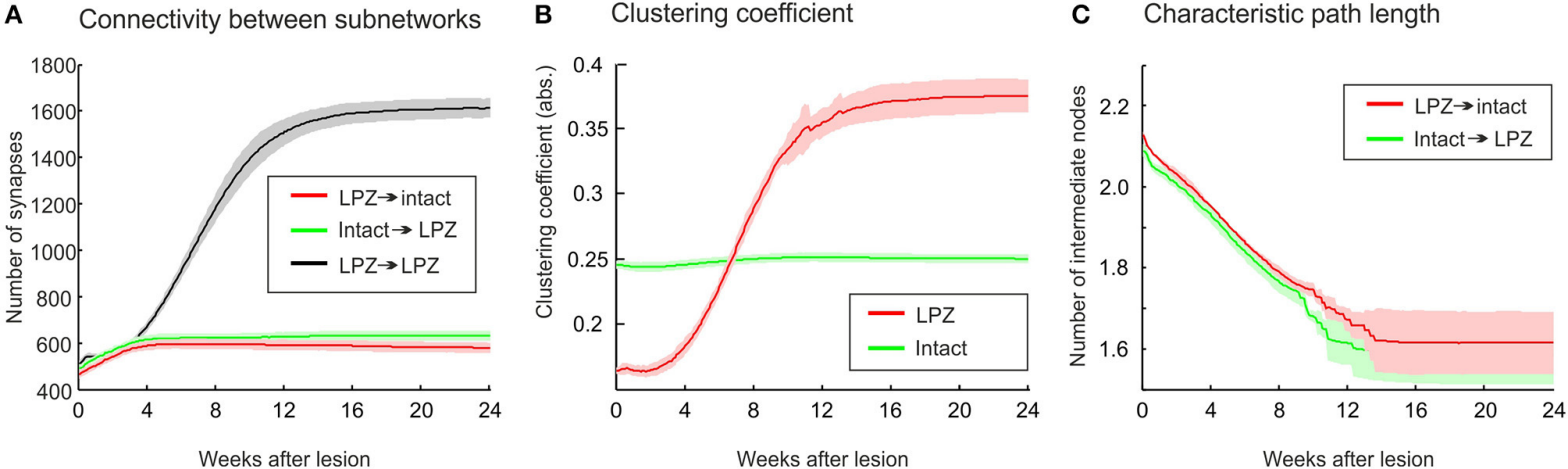
**The growth rules in the recurrent case, whereby axonal and dendritic elements can already form at low neuronal activity, have a considerable impact on network topology after the lesion. (A)** A strong increase in synapse numbers within the LPZ (black) is seen after the lesion. **(B)** The surplus of recurrent synapses in the LPZ gives rise to an increasing clustering coefficient of LPZ neurons (red) that even exceeds the clustering of the intact zone (green). In computing the average clustering coefficients over the excitatory neurons of the intact zone and the LPZ, we considered all excitatory connections from the entire network. **(C)** Average path lengths from neurons in the intact zone to neurons in the LPZ (green) and vice versa (red) show a steady decrease after deafferentation. Means over five simulations per scenario. Shadings of the curves indicate standard deviations.

The stronger increase in local efficiency in the recurrent case compared with the physiological case is brought about by the massive formation of partly recurrent connections originating from the LPZ. In fact, the number of recurrent synapses in the LPZ exceeds by far the number of synapses from the intact zone to the LPZ and from the LPZ to the intact zone (Figure 8A). The high number of recurrent synapses leads to a strong increase in clustering coefficient (Figure 8B). The clustering coefficient of the LPZ after rewiring even exceeds that of the intact zone; the latter does not change notably after the lesion. Remarkably, the average shortest paths from the intact zone to the LPZ and those from the LPZ to the intact zone strongly decrease simultaneously (Figure 8C).

### 3.3. Changes in degree distribution resulting from network rewiring

**Figure 9:**
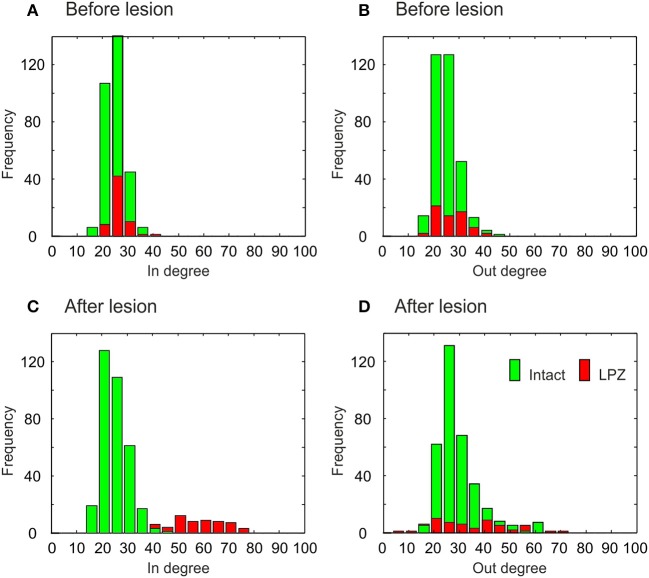
**Degree distributions of intact zone (green) and LPZ neurons (red) before and after the lesion in the physiological case**. In the bar charts, the larger amount is always placed in the background and the minor amount in the foreground. Before the lesion, the in-degree **(A)** and out-degree distributions **(B)** are not tailed. After the lesion, the distributions of in-degree **(C)** and out-degree **(D)** become more tailed. The diagram shows data from one simulation at *T* = 7950 and *T* = 20000 updates in connectivity, the latter corresponding to 24 weeks after lesion.

**Figure 10:**
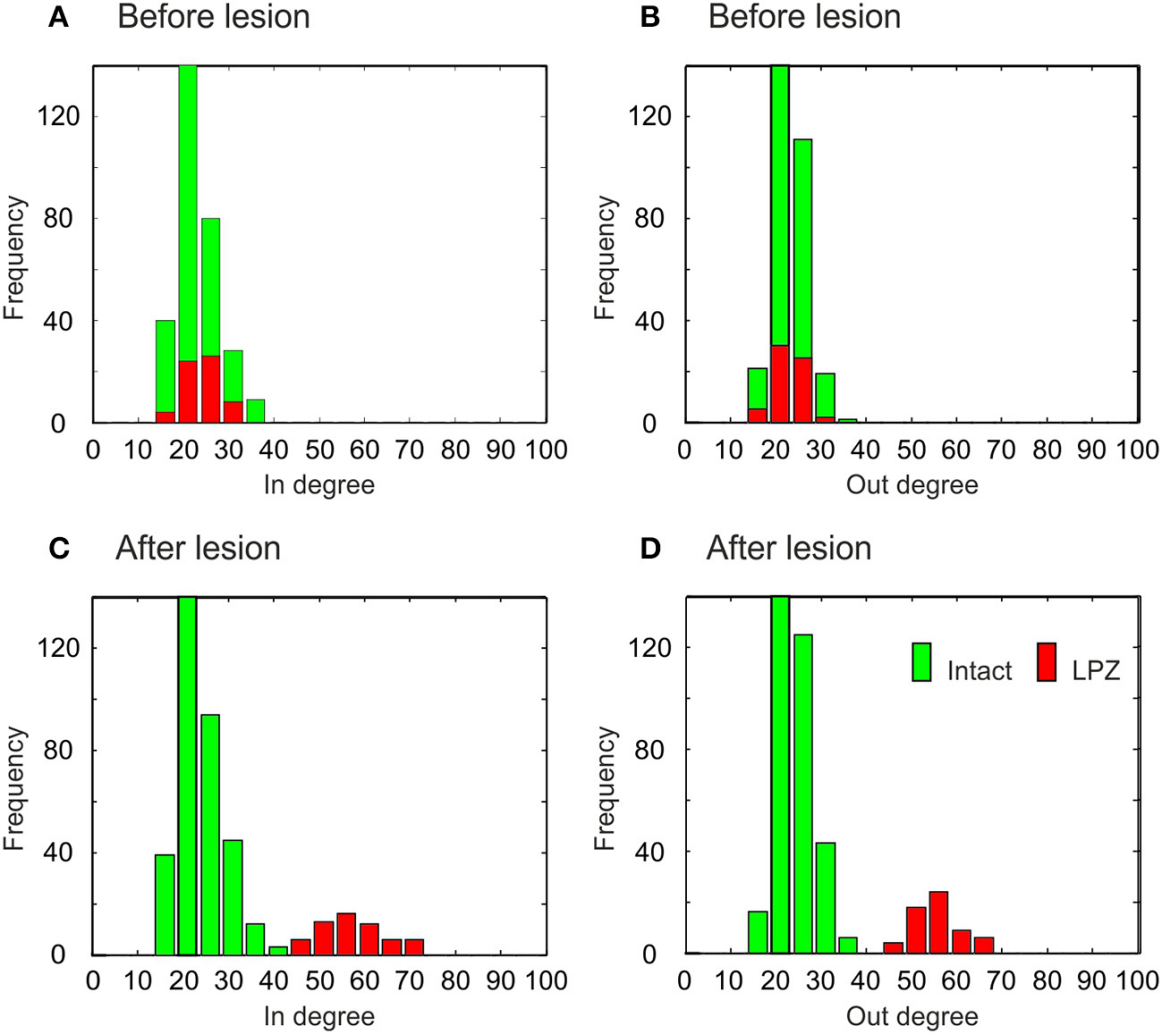
**Degree distributions of intact zone (green) and LPZ neurons (red) before and after the lesion in the recurrent case**. In the bar charts, the larger amount is always placed in the background and the minor amount in the foreground. Before the lesion, the in-degree **(A)** and out-degree distributions **(B)** are not tailed. After the lesion, the shapes of in-degree **(C)** and out-degree **(D)** distributions do not change markedly, but the centers of the in- and out-degree distributions of the LPZ neurons are shifted to the right. The diagram shows data from one simulation at *T* = 7950 and *T* = 20000 updates in connectivity, the latter corresponding to 24 weeks after lesion.

The different types of network rewiring have a direct impact not only on global network topology but also on the local degree distributions of neurons. Before the lesion, neurons of the intact zone and the LPZ have the same in- and out-degree distribution, in the physiological case (Figures 9A,B) as well as in the recurrent case (Figures 10A,B). The distributions in the physiological case are slightly more tailed than in the recurrent case. After the lesion in the physiological case, the center of the in-degree distribution of the LPZ neurons shifts to the right (Figure 9C), indicating the presence of neurons with high in-degrees. The centers of the out-degree distributions of LPZ and intact zone neurons do not change, but the distributions as a whole become more fat-tailed (Figure 9D). Thus, compensatory network rewiring generates more hub-like neurons in the LPZ, but the majority of neurons in the LPZ and the intact zone maintains its out-degree. In the recurrent case, LPZ neurons shift the centers of their in- and out-degree distributions completely to the right (Figure 10), but the distributions do not become more fat-tailed. The in- and out-degree distributions become markedly different from the ones before the lesion and from the degree distributions of the intact zone neurons. We may conclude that due to the massive recurrent connections, the LPZ neurons separate from the intact zone in terms of degree distribution.

### 3.4. Impact of initial topology on network repair

**Figure 11:**
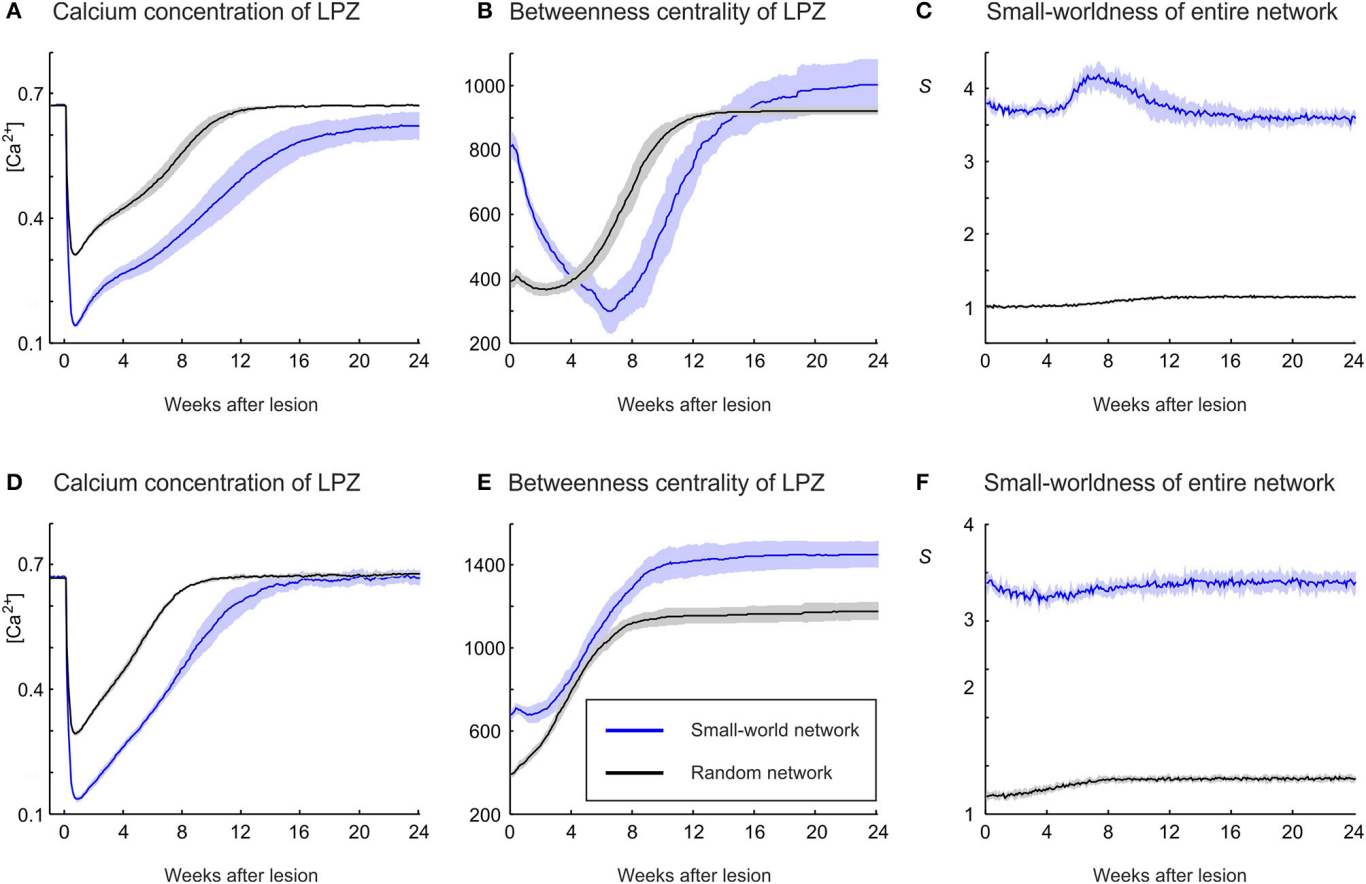
**The strong increase in betweenness centrality of LPZ neurons during network repair in the physiological case (A–C) and the recurrent case (D–F) is also obtained if the initial network topology is random. (A)** In the physiological case, random networks (black) restore activity levels after the lesion in a comparable manner to small-world networks (blue), but because levels do not drop as far as in small-world networks, random networks restore activity earlier than small-world networks. **(B)** The increase in betweennes centrality is obtained for random as well as small-world network topologies. **(C)** Random networks become slightly more structured after the lesion, as indicated by a small increase in small-world parameter *S*. **(D)** In the recurrent case, random networks restore activity levels after the lesion in a comparable manner to small-world networks, but because levels do not drop as far as in small-world networks, random networks restore activity earlier than small-world networks. **(E)** Betweenness centrality markedly increases in random networks, but to lower values than in small-world networks. **(F)** A small increase in small-world parameter *S* is also seen in the recurrent case with initial random topology. Means over five simulations per scenario. Shadings of the curves indicate standard deviations.

Network repair is not dependent on a particular initial network topology. The networks considered so far have a high clustering and a low characteristic path length before the lesion (small-world networks). However, even random networks with low initial clustering and characteristic path length fully restore their average electrical activity (in terms of calcium concentration) back to the homeostatic range, regardless of whether growth rules of the physiological case (Figure 11A) or the recurrent case (Figure 11D) are used. In random networks, the activity of the LPZ does not decrease so strongly as in clustered networks, because vacant axonal elements are available from anywhere in the network and network repair is immediately effective. The fastest restoration of electrical activity is seen for the recurrent case with random networks (Figure 11D). In addition to the availability of axonal elements from anywhere in the network, in the recurrent case neurons with low activity also provide their own vacant axonal elements, contributing to fast network repair.

Irrespective of growth rules and initial network topology, restoration of firing rates is accompanied by an increase in betweenness centrality (Figures 11B,E). For the physiological and the recurrent case, betweenness centrality reaches higher absolute values in small-world networks than in random networks. However, the greatest increase in betweenness centrality is seen for the recurrent case with random networks. Interestingly, for all scenarios studied (Figures 11A,D), the strongest increase in betweenness centrality is associated with the fastest restoration of electrical activity. We conclude that the increase in betweenness centrality is a generic effect of compensatory network rewiring because it is independent of initial connectivity and strongly correlates with effectiveness of network repair, in terms of speed and completeness of restoring electrical activity. Moreover, the physiological case with small-world networks is the only scenario in which topology becomes more random (Figure 11C). In all other scenarios (Figures 11C,F), we see only little change and initially random networks become only slightly more structured (small increase in *S*) after the lesion.

## 4. Discussion

We postulated that network repair after focal deafferentation is brought about by a local neuronal mechanism that aims to maintain homeostasis of neuronal electrical activity by adapting the neuron's number of input and output connections (homeostatic structural plasticity). In the model in which we studied the implications of this mechanism for network topology after deafferentation, we found that local changes in number of synaptic connections, as governed by homeostastic structural plasticity, led to pronounced alterations in global network topology, especially in the connectivity between intact and deafferentated areas. While local connections in the LPZ were massively eliminated, new connections from the intact zone grew into the LPZ, helping deafferentated neurons to restore their level of activity (see also Butz and van Ooyen, 2013). This replacement of short- by long-range connections lowered the clustering coefficient and reduced the characteristic path length, making the network more random than before the lesion. At the same time, neurons in the LPZ enhanced their betweenness centrality. Furthermore, LPZ neurons increased their global but decreased their local efficiency and got longer tailed degree distributions, indicating the emergence of hub neurons.

So far, only very few models have addressed dynamic changes in network topology after brain lesions. Li et al. (2013) described changes in topology merely phenomenologically and did not include any neuronal mechanism such as the formation and deletion of synapses. Others have applied neural mass models with various rules of plasticity and assessed by graph theoretical methods the changes in inter-area connectivity in response to lesions and degeneration (Rubinov et al., 2009; Stam et al., 2010; de Haan et al., 2012). In contrast with these more abstract models, our neuronal network model is more detailed and strongly inspired by the notion that neurons after a permanent loss of input, e.g., after focal retinal lesions, aim to restore their firing rates homeostatically by morphological adaptations such as the replacement of dendritic spines and axonal boutons. Therefore, we can derive predictions on how morphological alterations in individual neurons rewire intra-area connectivity in response to lesions or lasting loss of input. Insight into intra-cortical topology changes after loss of input is particularly important because local topographic features influence restoration of vision in humans (Sabel et al., 2011, 2013; Gall et al., 2013).

As yet, there are no experimental studies on dynamic changes in intra-area or inter-area network topology after focal retinal lesions, the experimental paradigm our model is most closely linked to (cf. Butz and van Ooyen, 2013). However, massive rewiring of synaptic connections not only occurs after focal retinal lesions in the visual cortex (Keck et al., 2008; Yamahachi et al., 2009; Marik et al., 2014) but also accompanies functional recovery after focal or subcortical stroke (Carmichael, 2003, 2006; Cramer, 2008). The findings from our model provide useful predictions also for focal or subcortical stroke, because the way a subcortical stroke affects cortical motor networks is essentially a deprivation of inputs from the lesioned subcortical to the intact cortical motor areas. Indeed, brain regions deafferentated by stroke show a restoration of electrical activity to normal levels in chronic patients, as measured by fMRI, that go along with persistent changes in inter-area topology (Sharma et al., 2009). We hypothesize that the, as yet not investigated, lesion-induced topology changes in intra-area connectivity may follow the same underlying rules as the observed changes in inter-area connectivity after focal stroke (Wang et al., 2010).

Lesion-induced structural plasticity does not always lead to restoration of impaired functions, and miss-wiring of brain circuits after lesions may even give rise to post-traumatic epilepsy (Topolnik et al., 2003). An additional interesting outcome of our model is that homeostatic structural plasticity can over-compensate a loss of input, resulting in pronounced oscillatory network activity that may account for the emergence of post-traumatic epilepsy (Butz and van Ooyen, 2013).

### 4.1. From micro- to macro-scopic

Remarkably, network reorganization in the model shows striking similarities with intracortical network reorganization on a mesoscopic scale [e.g., a retinotopic remapping with filling of the LPZ from the outside to the inside (Butz and van Ooyen, 2013); mesoscopic defined as in Liljenstroem, 2001] and may also account for macroscopic network changes after, for example, focal, subcortical stroke. An impairment of motor function of the hand after subcortical stroke coincides with a loss in effective connectivity of inter-area cortical motor networks, especially between pre-motor and primary motor cortices in the hemisphere ipsilateral to the stroke site (Grefkes et al., 2008). Conversely, restoration of electrical activity and functional recovery are associated with increasing effective connectivity from prefrontal to motor cortices (Sharma et al., 2009). The functional effects are thought to arise from a loss of local connections within the motor network and the formation of additional long-range excitatory connections from prefrontal to motor areas (Sharma et al., 2009). In our model, we also observe the local removal of connections from the deafferentated neurons in the LPZ and an ingrowth of more long-range connections from the intact zone into the LPZ. Note that in the model as well as in the brain, the loss of local connections is not a mere consequence of degeneration caused by the primary lesion but a secondary effect due to network reorganization (Rehme et al., 2011).

In stroke patients, even brain regions remote from the lesion site change their topology (Carmichael, 2006). However, not the entire brain changes its topology but only those regions directly connected to the primary lesion site (Carmichael et al., 2001; Dancause et al., 2005; Rehme and Grefkes, 2013). Provided that connected brain regions become deafferentated by the primary lesion site, homeostatic structural plasticity, as revealed by our modeling study, may account for the observed changes in macroscopic topology after a lesion, i.e., an increased randomness of network connectivity and an increased or decreased betweenness centrality of particular regions (Wang et al., 2010; Shi et al., 2013). Wang et al. (2010) reported an increase in betweenness centrality of brain regions that became deafferentated by a subcortical stroke, namely the ipsilesional primary motor area and the contralesional cerebellum. Note that the predominant cortico-ponto-cerebellar fiber tract crosses in the brainstem to the contralateral side, and a subcortical lesion will therefore deafferentiate the contralesional cerebellum. By contrast, the contralesional primary motor cortex and the ipsilesional cerebellum decreased their betweenness centrality. The latter two regions are not directly affected by deafferentation but are still involved in reorganization, since especially contralateral areas seem to support their homotopic regions by compensatory sprouting during stroke recovery (Carter et al., 2010). An increase in betweenness centrality of deafferentated brain regions and a decrease in betweenness centrality of brain regions supporting recovery perfectly match with our model findings, so we hypothesize that the observed topological changes in the brain of stroke patients may be accounted for by homeostatic structural plasticity. Furthermore, in the model, the increase in betweenness centrality has proven to be the strongest indicator of network repair under different conditions. Therefore, increasing betweenness centrality could be a biomarker for brain repair after lesions such as stroke. In future work, we intend to implement homeostatic structural plasticity in a large-scale model of micro- and macroscopic connectivity containing multiple brain regions (Potjans and Diesmann, 2014).

### 4.2. Time course of network repair

The time course of changes in topology in our self-repairing network model shows remarkable similarities with the time course of topology changes during brain repair, especially in patients with subcortical stroke. Subcortical stroke involves, apart from damage to a circumscribed volume of brain tissue, a loss of input to other brain regions, particularly those of the motor network. The model predicts a pronounced increase in small-worldness of the entire network during the initial phase of compensatory network rewiring, before the network in the end becomes more random. Indeed, brain networks after subcortical stroke increase their small-world property in the subacute phase (about 1 week post-infarct) (van Meer et al., 2012) and thereafter become continuously more random. As in our model, the change in small-worldness of brain networks is brought about by a marked change in clustering.

From our model we further predict that right after the lesion, local as well as global efficiency drops markedly as a result of loss of connections. The decrease in efficiency is in agreement with changes in network topology observed after stroke (Honey and Sporns, 2008; Alstott et al., 2009). In the model, local efficiency remains always lower than before the lesion, but global efficiency increases markedly and reaches values higher than before the lesion. Strikingly, even in well-recovered stroke patients, brain networks are found with low local but high global connectivity (Rehme and Grefkes, 2013). However, brain network topology with low local and high global efficiency may contribute to less stable performance of sensorymotor skills (Rehme and Grefkes, 2013).

Interestingly, in the model we observe only small changes in topology within the first 4 weeks. Network repair in stroke patients is also not immediate. From monkey studies it is well known that it takes about 7–14 days after stroke until axonal sprouting occurs, and new connections are visible not before 28 days (Carmichael, 2003), with lesion-induced network rewiring continuing for at least 3–6 months (Carmichael, 2006; Cramer, 2008). The time course of axonal sprouting in the experiment is comparable to the time course of axonal element formation in our model and also matches the physiological time course of structural plasticity in mice (Keck et al., 2008; Butz and van Ooyen, 2013). The model illustrates that network repair after deafferentation and stroke can be brought about by local, homeostatic growth rules.

The time course of network repair is determined by the relation between the growth curve parameters η_*A*_ and η_*D*_. If axonal elements require more activity to form than dendritic elements (i.e., η_*A*_ > η_D_), networks will show a compensatory growth of connections from the intact zone into the LPZ. However, if axonal and dendritic elements grow at the same low level of activity, deafferentated neurons will literally pull them selves by their own bootstraps by forming massive recurrent connections to restore activity to the homeostatic set-point (Butz and van Ooyen, 2013). By contrast, the course of reorganization is not crucially dependent on the particular choice of parameters for neuronal electrical activity (Figure S1), as long as the network is able to reach a homeostatic equilibrium before the external input is removed. Other parameters, such as the width of the kernel σ = 150 μm, have been chosen in agreement with experimental findings (De Paola et al., 2006). Likewise, the decay time of intracellular calcium was chosen to be of the same order of magnitude as measured experimentally (Hofer et al., 2011).

### 4.3. Homeostatic structural plasticity vs. synaptic scaling

The notion that neurons strive to restore their level of electrical activity after loss of input is now widely accepted (Hengen et al., 2013; Keck et al., 2013). Even in stroke, the need of neurons to restore electrical activity to a homeostatic set-point may be an underlying principle of recovery (Avramescu et al., 2009). Today, the predominantly discussed mechanism for maintaining homeostasis in electrical activity is synaptic scaling (Turrigiano and Nelson, 1998). However, synaptic scaling restores activity within 48 h, yet network reorganization continues massively for several months. Therefore, synaptic scaling cannot be the only mechanism involved in network reorganization after deafferentation and stroke. Moreover, Hengen et al. (2013) showed that firing rates in V1 after focal retinal lesions restore within the first 48 h but drop again thereafter before they slowly rise again. This finding is in line with previous reports on the extended time course of network repair (up to 12 months) after focal retinal lesions and the restoration of electrical activity from the outside to the inside of the LPZ (Giannikopoulos and Eysel, 2006). In the first 48 h after the lesion, homeostatic synaptic scaling may upregulate firing rates (Keck et al., 2013), but the continuing structural changes in connectivity beyond 48 h may alter activity levels and may bring neurons again outside their homeostatic range of activity. Rewiring connectivity may provide a straightforward explanation for the experimental observation that activity levels drop again after 48 h (Hengen et al., 2013) and slowly recover over several weeks (Giannikopoulos and Eysel, 2006; Hu et al., 2009, 2010). In the model, the removal of connections is accounted for by the minimal levels of activity needed for maintainance and formation of axonal and dendritic synaptic elements (η_*A*_ and η_*D*_, respectively); required minimal levels of activity have not been discussed in recent concepts of homeostatic plasticity (but see van Ooyen et al., 1996). In our model as well as in visual cortex after focal retinal lesions, activity slowly recovers over periods of weeks and months (Giannikopoulos and Eysel, 2006; Butz and van Ooyen, 2013). We hypothesize that the increase in firing rates is due to the ingrowth of long-range connections from intact regions, which may be guided by homeostatic structural plasticity. Therefore, we postulate that homeostatic plasticity is more than just synaptic scaling and needs to be extended to encompass structural plasticity, including the reorganization of synaptic connections. With the present model, we have provided growth rules that can govern homeostatic structural plasticity and that can lead to physiologically realistic network reorganization on a microscopic, mesoscopic (Butz and van Ooyen, 2013), and macroscopic level.

### 4.4. A potential role of homeostatic plasticity in epileptogenesis

Partial deafferentation, as caused by focal stroke for example, can lead to epileptiform activity and seizures (Topolnik et al., 2003; Avramescu and Timofeev, 2008). It has been discussed that homeostatic synaptic plasticity may contribute to post-traumatic epileptogenesis in chronically isolated cortex (Houweling et al., 2005). Synaptic scaling (Turrigiano and Nelson, 1998), a well-studied mechanism for homeostatic synaptic plasticity, is known to generate epileptiform activity (Froehlich et al., 2008). However, synaptic plasticity does not include the rewiring of networks and acts on timescales of hours rather than weeks or months. Although a previous modeling study (Houweling et al., 2005) has suggested that anatomical network rewiring is not required for epileptiform activity to occur, we argue that without network rewiring an important aspect of lesion-induced plasticity is left out. For example, models without structural plasticity cannot account for the clinical observation that although spontaneous seizures are most frequent within months after the lesion, they can occur up to 5 years post-lesion (Temkin, 2001). Therefore, we propose that synaptic scaling may account for spontaneous seizures early after the lesion but that for the pathogenesis of post-traumatic epilepsy months after the lesion, homeostatic structural plasticity may be a more suitable explanation (see also van Oss and van Ooyen, 1997).

In the model, a change in the value of just a single parameter, namely the level of activity needed for axonal elements to form (η_*A*_), leads to massive recurrent connections, which, as we showed in a previous study (Butz and van Ooyen, 2013), can generate strongly synchronized activity patterns comparable to epileptiform activity. In an *in vitro* injury model of epilepsy, Srinivas et al. (2007) showed that epileptogenesis goes along with a marked increase in connectivity [also supported by findings on recurrent mossy fiber sprouting in an organotypic cell culture model of hippocampal epilepsy (Kharatishvili et al., 2007)] and that the shape of the degree distribution of the neurons changes from power-law to Gaussian. Interestingly, in the recurrent case of our model, which generates epileptiform activity after network reorganization, the degree distribution of LPZ neurons is much less tailed than in the physiological case after network repair. Therefore, we hypothesize that the way brain networks rewire after lesions determines whether or not patients develop post-traumatic epilepsy. This notion is further supported by the finding that the shape of the lesion can affect epileptogenesis (Volman et al., 2011), since it is more likely that the shape of the lesion can influence epileptogenesis with growth of new connections than with synaptic scaling. Importantly, our model predicts that the sensitivity of axonal outgrowth to low levels of activity might be decisive for whether recurrent connections with epileptiform activity, or physiological network repair with normal activity patterns, emerge after brain lesions. This insight may help find novel molecular targets for pharmacological treatments to prevent post-traumatic epilepsy, which are urgently needed as post-traumatic epilepsy is often impervious to medical treatment (Herman, 2002; van Breemen et al., 2007).

### 4.5. Homeostatic structural plasticity as an organizing principle for brain repair

Homeostatic structural plasticity is a new concept for network reorganization, with large implications for understanding and stimulating brain repair after lesions. Models of homeostatic structural plasticity can help integrate recent clinical findings on changing brain topology after a variety of pathologies, including stroke, Alzheimer's disease and multiple sclerosis. These models can assist us in uncovering the mechanisms underlying functional reorganization and in finding biomarkers for successful brain repair, such as an increased betweenness centrality of brain regions deprived of input from primary lesion sites. Most importantly, however, homeostatic structural plasticity puts functional reorganization of brain networks into a different light. The predominant dogma of plasticity is still Hebbian plasticity, with its “fire together, wire together” slogan (Hebb, 1949). With Hebbian plasticity, enforcing (synchronous) activity strengthens synapses. By contrast, the homeostatic nature of structural plasticity implies the need for a moderate level of activity, because the formation of axonal and dendritic structures is maximal for activity levels slightly below a desired set-point of electrical activity. We postulate that the brain has the highest plasticity for recovery when neurons and brain regions, especially those supporting deafferentated regions in the recovery process, have not yet returned to their homeostatic equilibrium. We might call this initial phase a critical period for brain repair, in analogy to critical periods in neural development. During network development, too, neurons shape their connectivity until desired activity levels are reached (Tetzlaff et al., 2010). As a consequence, in neurorehabilitation treatment, not only stimulation by physical training or direct electrical stimulation but also pauses in treatment may be important. Stimulation may increase electrical activity beyond the homeostatic set-point, inducing pruning of existing synaptic connections, whereas treatment pauses may lower activity and bring activity levels into an optimal range for the formation of new connections (Butz et al., 2009a). Moreover, network reorganization does not always need to be functional; as our model suggests, post-traumatic epilepsy could be the result of miss-wiring or over-compensation. Treatments must therefore focus more on the time course and current state of network repair. Lastly, large-scale computer models, such as those developed in the context of the human brain project (www.humanbrainproject.eu) will, once structural plasticity has been incorporated, be valuable tools in finding and testing treatment strategies for patients with brain damage.

### Conflict of interest statement

The authors declare that the research was conducted in the absence of any commercial or financial relationships that could be construed as a potential conflict of interest.
