# Supplementary material for: Homeostatic structural plasticity can account for topology changes following deafferentation and focal stroke
Source: Front Neuroanat. 2014 Oct 16;8:115. doi: 10.3389/fnana.2014.00115 (PMC4199279; doi:10.3389/fnana.2014.00115)
Supplement: Supplementary file 1 [file Image1.PDF]

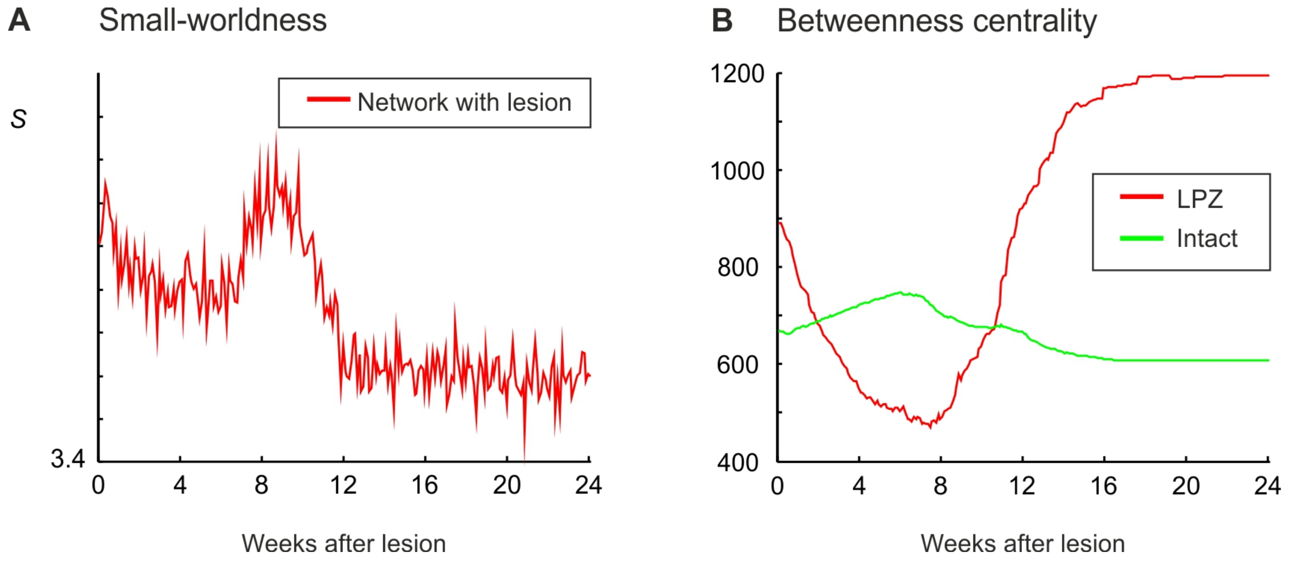

Figure S1. Comparable changes in network topology can also be obtained with different neuronal dynamics. For this simulation, we used the following parameters in the Izhikevich neuron model (Eq. 1): $a = 0.1mV^{-1}$, $b = 0.2mV^{-1}$, $c = -55mV$, $d = 4mVms^{-1}$, which produced neuronal firing with intrinsic bursting. The other parameters, including the growth parameters, were the same as in the physiological case. A) Also with this choice of neuron parameters, we observe that the network first increases its small-worldness and subsequently decreases it below levels before the lesion. Thus, the network becomes more random as a consequence of network rewiring. B) At the same time that the network as a whole becomes more random, neurons in the LPZ increase their betweenness centrality, while neurons in the intact zone decrease their betweenness centrality. Compare with Figure 2B,C.
